# Society Notes

**Published:** 1901-07

**Authors:** 


### Society Notes.

### Association of Health Officers of Texas.

[Meeting in Austin, Texas, March 21, 1901—continued.]

The following papers were read at the March meeting of the Health Officers' Association:

(The next meeting will take place at Dallas, August 2, prox.)

### Smallpox or Cuban Itch?*

W. H. BLYTHE, M. D., COUNTY HEALTH OFFICER TITUS COUNTY, MT. PLEASANT, TEXAS.

Just a few words as a preliminary. I wish to call your attention to the position I take in the following article. I have tried to imagine myself a real Cuban itch advocate and believer, and I can tell you now it has been the hardest job I ever did. I do so that you may see the status of opinion and acts of some merchants, doctors and even officials, also many who wish to be on the side of those Neros whom an honest serf or slave would detest and loathe, because they can see the slain, disfigured and wrecked bodies of innocent people, almost every day, through the machination of these murderous smuggling machiavilians. Showing not so much consideration for the protection of the people as the Tartar poten-

*Read at meeting of State Association of Health Officers, March 21, 1901.

tate, Timon, did; for all he builded bridges out of human bodies and pyramids out of human skulls.

Bunyan had an active imagination; Washington Irving had to correct some of his imaginations; many of the Spanish explorers were deluded into severe straights by their imagination; historians have been misled by imagination; Columbus was a victim of imagination; Pascal was a great sufferer from his peculiar imagination; Poet Shelley had a hard time with his overwrought and diseased imagination, and Napoleon said "Imagination rules the worlds." But Napoleon nor any one else ever had such a job of imagination as it would be to imagine that a disease having all the clinical history of smallpox was Cuban itch.

However, away back in early times when but little more than the first half of the fifth Christian century had gone into history, and about a quarter of a century before Mahomet had his first bellyband adjusted, there was an outbreak of an eruptive disease called Cuban itch, in and around Pelusium, Egypt, from which place it spread to Turkey.

Paulus Agineta wrote a great deal on medicine and surgery some thirteen hundred years ago, and in all of it he did not say one word about smallpox. Rhazes, eleven centuries ago, was considered one of the best, most competent and accomplished writers on medicine and diseases; and he gave us an unmistakable description of Cuban itch, besides he spoke of other medical men of Alexandria and Bagdad who wrote about Cuban itch.

Cuban itch is a coveted disease, it is sought for harbored and secreted, as it seems, by many voters, either through ignorance or pure cussedness, in some States, especially in some parts of Texas. I say coveted for the very reason that there are so many who, if not at present, have in the recent past, expressed themselves as being desirous of having it, if that is what the State Health Officer—a political appointee—calls smallpox, and to have it will protect us from smallpox, should it ever make its appearance here in our midst. Well, we want it, and we ought not try to hinder its progress, it don't hurt anybody, and then besides it is nearly altogether among the negroes and the insanitary whites. Yes, it seems to be a catching disease too, as it catches lots of negroes and some of these insanitary white folks, as catlike it begins to get out its claws and get on its catching clothes about cotton-picking season and don't get through catching during the winter and plow-time in the spring, and even lurks around all through planting and on into the watermelon season, before it gets tired; then it is in hiding only as is to be plainly realized, for it appears to be ready and on hand early in the fall so as to aid in lessening the number of cotton pickers and shortening the cotton output—that is, judging from the experience of the last two years.

Cuban itch, chickenpox, impetigo contagiosa and the rash are great friends, and have a considerable number of followers among American Aesculapians. The former is likely to make its appearance in epidemic form, as is proved by its characteristics during the past two years. Its advent into the United States is said, by those who know, to be through some Americans who went over to Cuba and mixed up with the Cubans, then when they returned to their native land it was discovered by the Provost Medical General, and lots of the rest of us medicos, that they had introduced to our notice a new disease, and we at last began to believe it was catching, as it got scattered so. That it is an epidemic disease is evidenced by the way it grew to such huge proportions, as from a few Americans it rose up and scattered itself or expanded over forty-four States and Territories in a few months, and settled down in spots as large as a county. Cuban itch is a political disease, of the gold standard type, and tends to and holds to the idea of expansion. It is in fact a true expansionist.

Voters, their wives, old maid sisters and bachelor brothers, are not quite so liable to it as young negroes and their white playmates; although people of all ages are not exempt, without they have a genuine vaccination. It not only attacks negroes and white people, but has been claimed by some merchants, not all, thank God, that it shows its greatest affinity and most distinct character and effects on trade.

The fact is so well established now that vaccination prevents Cuban itch spread many are resorting to it, especially when there is a little rash or Cuban itch in the neighborhood, at the next house or nearer. They carry vaccination so far as to use the virus in profusion and often. We firmly believe that vaccination is to Cuban itch what the Chinese wall was to the wild Tartars.

It is rare for a person to have a second attack, yet there are instances where the second attack has occurred, and even the third. "If that is smallpox, we want it! If it should occur in my house, I would conceal it and not devulge it until I had to!" My friends, neighbors or customers, if you should happen to be afflicted with this rash that these health officers call smallpox, why, pay no attention to it, come right on and get your flour and calico. We don't believe in any such stuff—its only a money scheme. Who's afraid? There is no smallpox here now, nor has there been. I will eat all the scabs you can get in the county that come from genuine smallpox cases. I think it is a shame for the commissioners court to allow themselves to be imposed upon in any such way.

The Provost General of the Marine Horse Show of a great Western Republic once sent, as a foot note, in plain language, telling all about the origin of Cuban itch, but we know some things ourselves. We know that if he had read as much literature about Cuban itch as he has quarantine and smallpox, no doubt it would have made a very material change in his knowledge and opinion. Besides, he surely has not consulted the mercantile interests as much as Cuban itch people have and do, who know Cuban itch, rash, chickenpox, impetigo contagiosa, etc.

I have seen a whole lot of Cuban itch during the past two years, so I ought to know it when I see it, and claim to. Why, it can be so very mild that a negro having it can walk along the street and into a store, step up to a counter and be attended by a white lady clerk, who don't observe anything wrong with the customer. This same Cubanola will go home and attend to the house work of some white family, and nothing disturbs their quiet for several days, when one or more of the family take a high fever and severe headache; the doctor is called, and he prescribes, and continues his visits to the patient for two or three and now and then four days, when the fight is believed to be won, as the fever and headache subsides, and the patient about easy. So the case is dismissed, but advised to continue the quinine a day or two longer, as they only had a little bilious attack aggravated by the association of a touch of the grip. When to the surprise of the family, next day, there are some little hard pimples showing in or beneath the skin, anyway they can feel something hard and they keep getting larger and begin to itch. Well! well! what is this? Go tell the doctor that Mimy has broken out all over, nearly, with little hard bumps, and come at once. It so happens that the doctor's olfactory organ is very sensitive to all odor of the mus musculus, and having a very acute attack, can't go, but sends all necessary instructions, and the messenger too. Of course, we know it is, perhaps, a little rash and possibly it may be slightly catching, though we are not afraid to go, yet we have patients who are a little whimsical and rather we wouldn't go, so just to satisfy both the parties we don't go only to the "phone" in the hall—our hall—but it is not smallpox all the same.

So that case and many others are treated clandestinely and by telephone between suns. That shows you plainly how much more popular we Cuban itch "fellers" are with those who are too high in the social scale to have what you State, county and city health officers call smallpox. We defy your State statutes.

There are those who are not so fortunate, for they take a very high fever, excessively severe headache, backache and in fact ache like they had wrestled with Hector before the gates of Troy, and old Priam had beat them while Hector held them down. Now this state of affairs, with perhaps some little variation, continues for two, three or four days, and during this time they load up on calomel, quinine, fever and headache powders, but as a rule on the third day the fever drops to normal or nearly so, headache about all gone, together with most all the chilly sensation, and feeling pretty fair. It is not necessary to tell you about the respiration, eyes, throat and how thirsty they, in most instances, become; also the white furred tongue, and a full realization of a disgusting sensation between the ensiform cartilage and the navel, before relief is obtained. Scratching has already been in progress a few hours, and will continue to a greater or less degree for some days, but it is possible to lessen it greatly by the proper use of 1-1000 international bedbug solution; in fact, it controls it. The scratcher along about this time feels some little hard bumps in or under the skin which, as the hours pass by, keep rising higher and higher, larger and larger, until they become real Cuban bumps, but they don't stop at that; they begin to soften on top and become white or yellowish white, as though there might be some fluid of that color in them, but still the bumps don't stop there; they keep going till the time comes to stop, and not before. By this time the scalp gets as thick as a cowhide in a tan vat, the forehead and cheeks look like the proprietor had been on a week's spree and the eyes were hiding behind the fullness thereof, and the feet require shoes, if worn at all, three numbers larger than before.

We are now somewhere between the seventh and eighth day of the eruption, and the bumps are about as full as they propose to get, and by reason of the character of the material within, these bumps are now called Cuban pus bumps or pustules, and about this time you will observe two conditions presenting themselves—one is the facial outlines are so changed, and to such a degree, the photographer or camera one won't take the picture, and the other is a peculiar odor. It is not very much unlike a fifty per cent. strength odor one detects as it is gently wafted on the evening breeze of a damp day from off a pile of burning cotton seed. It might be that this Cuban itch odor is due to exhalation from the skin of Cubanic acid.

After the pustules have reached maturity they begin to depress at the center, and from that point start on their dying trip, no matter whether they rupture of their own accord or you break their heads yourself. They form scabs which when they are ready—not you—fall off and leave spots that look like one who had been shot with a load of split fox grapes.

Right here I wish to record one thing I have observed that is peculiar to those mean mangey adult Cuban itch patients, and that is the frequent repetition of two very familiar religious hymns. During all the primary fever, headache, Hectorian soreness and aching, one particular hymn is sung, preceded by the exclamation of Oh! my head! Show pity, Lord! Oh! Lord forgive. When the headache and fever subsides and the bumps begin to appear, the Cubanola gradually changes his petitioning hymn into one of thanks, for instance, I am glad salvation's free, salvation's free for you and me.

The cause of Cuban itch is unknown except to some doctors, merchants and megacephalus-janus-scallawag-policyists, and we are not going to tell it, and as far as we are concerned the community can look to fate and Bibulus. We could without any impropriety or weakening our position admit that it is a specific disease; but we won't admit that Cuban itch is smallpox. You smallpox folks go on to describe four well marked varieties—with papular, vesicular and pustular developments, etc.—while we simply claim that Cuban itch is Cuban itch, and you can't make anything else out of it.

In conclusion, as I have been talking to doctors of the smallpox persuasion, I wish to say a few words about legislation as regards the Temple paper recommended for consideration by our Texas Legislature. I hope that every legislator may be able to see that such legislation is greatly needed, and in granting it, the very best interests of the people will be served, and without that for a law, and that right away, conditions will come about that will settle all this dispute, and that with a sad and heart rending experience to many of our good citizens before another twelve months has passed.

DISCUSSION.

Dr. Jones: "Dr. Blythe's paper marks an epoch in history; all will arise and call him blessed. He is the first man who has ever described the disease. The symptoms of the two diseases are similar, but Dr. Blythe has untied the knot. The diagnosis depends upon the point of view. The health officer will say 'smallpox' and the public will diagnose 'Cuban itch.'"

Dr. Grube called attention to the fact that many syphilitics were affected by smallpox.

Dr. Graves said he had treated over five hundred cases in the last two years, and had never seen a case of Cuban itch. He commended the commissioners court for so bravely assisting him in endeavoring to suppress the disease. He received the smallpox into his county from Washington county, and during the last two years has had three hundred cases. While may cases were mild, during the last month he had some very serious ones, three or four deaths having occurred.

Dr. McCutchan also received smallpox into his county from Washington county and stated that it was his opinion that the wide spread of the disease from that county was due to the fact that they paid no attention to it there, calling it Cuban itch, eruption after la grippe, etc. But if it be an eruptive disease, called by any of these names, vaccination is good for it. It is my opinion that immunity, from vaccination, is hereditary. Out of 207 cases that I have treated, only fifty-three were serious, and I hold this fact due to parental vaccination.

Dr. Massie: "Being health officer of one of the largest cities of the State, I have run across as much smallpox as any gentleman present. I have seen the 'itch' in Cuba. I was there with the regiment and volunteered my services in taking charge of the smallpox. After twenty-four days there were 250 patients and a death rate of thirty per cent., and within four or five months time 1500 cases with a death rate of seven per cent. This was to an extent due to the supreme authority of the militia. 'Cuban itch' is unknown in Cuba; we come to America to find it. However, in Houston, we consider it more complimentary to have smallpox than 'Cuban itch.'"

The following paper from Dr. Guinn was then read:

### Compulsory Vaccination.

DR. E. E. GUINN, COUNTY HEALTH OFFICER CHEROKEE COUNTY.

*Mr. Chairman and Gentlemen of the Association of Health Officers of Texas:*

In response to your invitation to meet with you in Austin on the 21st inst., or contribute a paper to be read at such meeting, I assure you that I regret very much my inability to attend, but tender you my hearty co-operation in your good work, and will say I know if the ideas of this body were only enacted into a law, that within five years there would not be a single case of smallpox to develop within the borders of this State in the person of a native Texan.

My ideas briefly stated are as follows:

First of all, compulsory vaccination to be repeated every five years, the State bearing the expense for the vaccine virus, and in order that this expense be reduced to a minimum, let her, upon the plains of some of her school lands, establish and maintain a State Chemical and Bacteriological Laboratory and Vaccine Farm, manufacture her own virus, as well as her diphtheria antitoxine and all other serums and antitoxins now in general use. These to be supplied to all county health officers for the free distribution to every physician in his county; and said physician to furnish gratis to every person in his territory (or locality), and this act carry with it a penalty of not less than $10.00 nor more than $100.00 to be assessed against every single individual, and every head of a family, who neglects, or refuses, to present himself or his family for vaccination. Let each physician vaccinate every infant born, at the age of thirty days, if the necessity demands, and not later than two years. Let the direction of this work be carried on by the county health officer, who shall be under the jurisdiction of the State Health Officer or State Health Board. This will clear Texas and keep her clear of smallpox, and no less stringent methods will ever do it.

### COUNTY HEALTH OFFICER AND HIS DUTIES.

Let there be appointed by the commissioners court, at their February term, a county health officer, who shall serve for a term of two years or until his successor shall qualify, and he should be selected from a standpoint of ability. He should possess executive ability in connection with his professional ability; he should be empowered to act independently of the commissioners court in an emergency, his acts to be ratified by the court at its next meeting. He should be equipped with disinfectants, apparatus, oil suit, record books, etc. He should be paid according to the population, towit:

| | |
|---|---|
| In counties having less than 10,000 inhabitants......... | $ 300.00 |
| Over 10,000 and less than 20,000...................... | 400.00 |
| Over 20,000 and less than 30,000...................... | 500.00 |
| Over 30,000 and less than 40,000...................... | 600.00 |
| Over 40,000 and under 50,000........................ | 700.00 |
| Over 60,000 ........................................ | 1000.00 |

This to be paid by the respective counties. I arranged this schedule according to taxation, and I put it small from the fact that county health officers would have little to do after the first five years.

Let each incorporated town select her city health officer at a fee of $10.00 per day while actually engaged in such work, and who shall work under the orders of the county health officer. This expense and all others connected with a quarantine and incidental thereto be paid by the State, except the county health officer's fee and equipment, which the county shall pay for.

Be it enacted that every physician failing to report to the county health officer any case of smallpox, varioloid, diphtheria, yellow fever or cerebro-spinal meningitis that he be fined not less than $50.00 nor more than $500.00, or imprisonment for not less than ten days nor more than six months, or both fine and imprisonment for such failure. Each county health officer to make immediate report of such cases to the State Health Officer or State Board.

Each county health officer shall make a quarterly report to the commissioners court, who shall approve his accounts, which must be itemized before presentation, and payment to be made quarterly by the State, with the exception of county health officers fees, which shall be paid by the county in which he is employed.

But, gentlemen of this convention, as lovers of humanity, we have more and graver questions confronting us today than all of the above mentioned diseases, and it is the great and awful destroyer of health and happiness and future expectations, of homes and of life —consumption.

Consumption is killing more people in the United States today than all the above mentioned diseases together. Is this not true? What can be done?

First, we can prevent many, yes, very many, cases; we can help some, and cure a few. First, we must educate the people to take proper physical exercise, and especially should the lungs have the proper attention, and sleeping apartments kept clean and well ventilated, and of course our bodies should always be kept clean. After all debilitating diseases we are more prone to contract consumption. Our vitality can not resist the enemy that is everywhere present; therefore, we should be more anxious about our patients during their convalescence, and especially those who have been suffering from catarrhal diseases of the respiratory tract, and low grades of continued fevers, and chronic diseases. Give them first of all plenty of pure, fresh air, sunlight and water, and some appropriate tonic, and impress them with the necessity of their continuance.

Let there be enacted a law with a penalty that every landlord be and is hereby required to immediately disinfect and sterilize after the removal of a tenant who has, while residing within said house, been afflicted with smallpox, measles, scarlet fever, whooping cough, mumps, typhoid fever, yellow fever, cerebro-spinal meningitis or consumption; this to be done under the supervision of the county health officer, if in reach, and if not, by his family physician. This measure alone will curtail considerably the number of consumptives.

Yesterday two cases of smallpox were reported to me, and on my arrival found that twenty or more people had been exposed, and half of this number refused vaccination, stating "that I would have to vaccinate their dead bodies," etc., and "that I would have to retain their dead bodies." Now, had we had on our statute a compulsory vaccination law, these people would readily have consented. And today a citizen came to me and reported a case of smallpox in his neighborhood—and in five miles of our town—and one of our town physicians treating the patient, and when I saw the physician in attendance he stated to me that I had been correctly informed by the citizen. He said "the patient is clear of fever and convalescing." He says I told them they need not quarantine, but to be vaccinated and stay at home. I shall go there tomorrow, and should there appear more cases it should justly be charged to the action of the physician in not reporting the case and having proper action taken.

Now, had we such a law as that herein indicated this physician would have been very prompt in making his report and thereby saved time and expense, and perhaps life.

Rusk, Texas, March 14, 1901.

